# Prevalence of Comorbidities in Asthma and Nonasthma Patients: A Meta-analysis

**DOI:** 10.1097/MD.0000000000003459

**Published:** 2016-06-03

**Authors:** Xinming Su, Yuan Ren, Menglu Li, Xuan Zhao, Lingfei Kong, Jian Kang

**Affiliations:** From the Department of Respiratory Medicine, Institute of Respiratory Diseases, The First Affiliated Hospital of China Medical University, Shenyang, China.

## Abstract

This study compares the prevalence rates of comorbidities between asthma and nonasthma control patients reported in the literature.

Literature was searched in several electronic databases. After the selection of studies by following précised eligibility criteria, meta-analyses of odds ratios were carried out with subgroup and sensitivity analyses.

Eleven studies studying 117,548 asthma patients compared with 443,948 non-asthma controls were included in the meta-analysis. The prevalence of cardiovascular comorbidities (odds ratio (OR): [95% CI] 1.90 [1.70, 2.14]; *P* < 0.00001), cerebrovascular comorbidities (OR 1.44 [1.29, 1.60]; *P* < 0.00001), obesity (OR 1.51 [1.14, 2.01]; *P* < 0.00001), hypertension (OR 1.66 [1.47, 1.88]; *P* < 0.00001, diabetes (OR 1.25 [1.08, 1.44]; *P* < 0.00001), other metabolic and endocrine comorbidities (OR 1.60 [1.40, 1.83]; *P* < 0.00001), psychiatric and neurological comorbidities (OR 1.62 [1.44, 1.82]; *P* < 0.00001), gut and urinary comorbidities (OR 1.91 [1.47, 2.49]; *P* < 0.00001),), cancer (OR 1.17 [1.10, 1.25]; *P* < 0.00001), and respiratory comorbidities (OR 5.60 [4.22, 7.44]; *P* < 0.00001) were significantly higher in the asthma patients in comparison with nonasthma controls.

Asthma is associated with significantly higher comorbidities including cardio-/cerebrovascular diseases, obesity, hypertension, diabetes, psychiatric and neurological comorbidities, gut and urinary conditions, cancer, and respiratory problems other than asthma. Respiratory comorbidities are found 5 times more prevalent in asthma than in non-asthma patients.

## INTRODUCTION

Asthma is a common inflammatory disorder of the respiratory tract, which is characterized by the obstruction and hyper-responsiveness of the tracheo-broncheal system.^1^ Repeated episodic shortness of breath with variable expiratory flow, wheezing, recurrent cough, excessive mucus production by the lining of air passage and chest tightness are the major symptoms of this disease.^2^ Asthma is recognized as a heterogeneous disease as multiple subtypes of this disease with distinct pathophysiologic mechanisms are identified.^3^

Estimates of asthma in elderly patients (>60 years of age) indicate 4% to 13% prevalence.^4^ Asthma is an important cause of morbidity and disability in individuals over 65 years of age as control of this disease worsens in later age, which is also associated with increased emergency medicine as well as hospitalizations.^5^

Comorbidities are increasingly recognized as important determinants of asthma management and prognosis as these are associated with inadequate disease control, higher health care use, and poor quality of life.^6^ Moreover, the recurrent exacerbations in asthma are associated with specific co-morbidities that require additional therapeutic interventions.^7^ In elderly, especially, the comorbidities are associated with higher mortality, poor adherence to therapeutic interventions, and reduced quality of life.^8^

Although a considerable number of studies have documented the prevalence of comorbidities in asthma patients, comparative controlled studies are relatively less in number. The aim of the present study was to undertake a systematic literature search and to perform a meta-analysis of the studies which compared the prevalence of comorbidities in asthma and nonasthma patients in order to examine the significance of difference in the prevalence of various comorbidities between asthma patients and nonasthma controls.

## METHODS

Preferred Reporting Items for Systematic Reviews and Meta-Analyses (PRISMA) guidelines^9^ are followed while performing this meta-analysis and associated systematic review. As this study is a meta-analysis research with the published data as materials, it does not need the approval from the institutional review board.

### Literature Search

Several electronic databases including Embase, Google Scholar, Ovid SP, Pubmed/Medline, and Web of Science were searched for the relevant articles. The major medical subject headings (MeSH) and keywords used in different logical combinations and phrases included asthma, comorbidity/comorbidities, prevalence, cardiovascular, heart disease, stroke, myocardial infarction, thrombosis, atherosclerosis, hypertension, diabetes, obesity, thyroid disease, skin disease, cancer, malignancy, psychiatric disorders, depression, neurological disorders, psychosis, respiratory conditions, gastrointestinal diseases, and musculoskeletal disorders. The search encompassed original research papers published before November 2015.

### Inclusion and Exclusion Criteria

The inclusion criterion was: studies reporting the prevalence of the comorbidities in asthma patients by comparing with suitable nonasthma control patients. The exclusion criteria were: (a) studies providing comorbidity prevalence data in asthma patients without control data or studies utilizing asthma/chronic obstructive pulmonary disease controls; studies utilizing indirect data such as medical claims; study reports with data in forms that were unable to be utilized in the meta-analyses of odds ratios.

### Data Extraction, Synthesis, and Statistical Analysis

Required data and corresponding demographics were obtained from the selected research articles and synthesized on spreadsheets for use in the meta-analyses. Meta-analyses were carried out with RevMan (Version 5.3; Cochrane Collaboration) software under fixed effects as well as random effects models. For the meta-analyses, the prevalence data were used to calculate the odds ratios of each study data and the overall effect sizes were generated, which was a weighted average of the inverse variance adjusted effect sizes of individual studies (odds ratios [OR] along with 95% CI). Between studies consistency was tested by the *I*^2^ index. Subgroup analyses were carried out and sensitivity analyses performed in order to explore the authenticity of results and sources of higher heterogeneity. Publication bias assessment was made by the visual examination of the funnel plot symmetry.

## RESULTS

**FIGURE 1:**
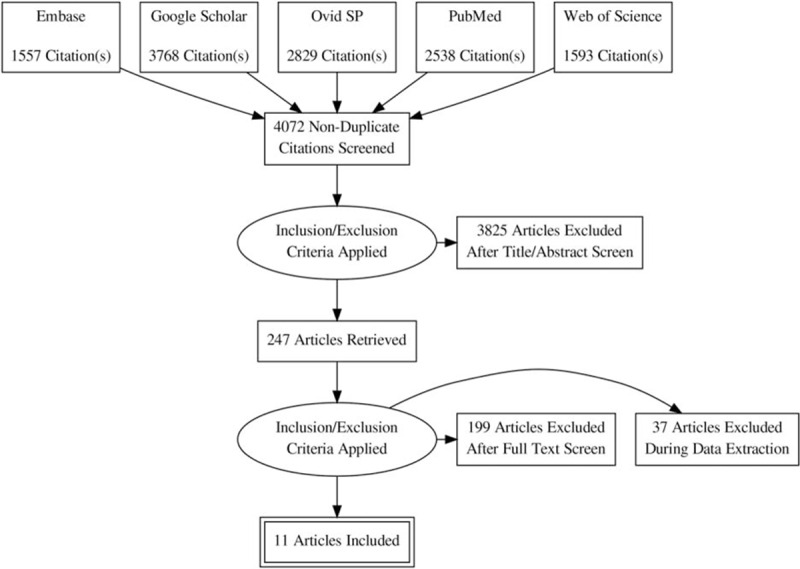
A flowchart of the literature search, study screening, and selection process.

**FIGURE 2:**
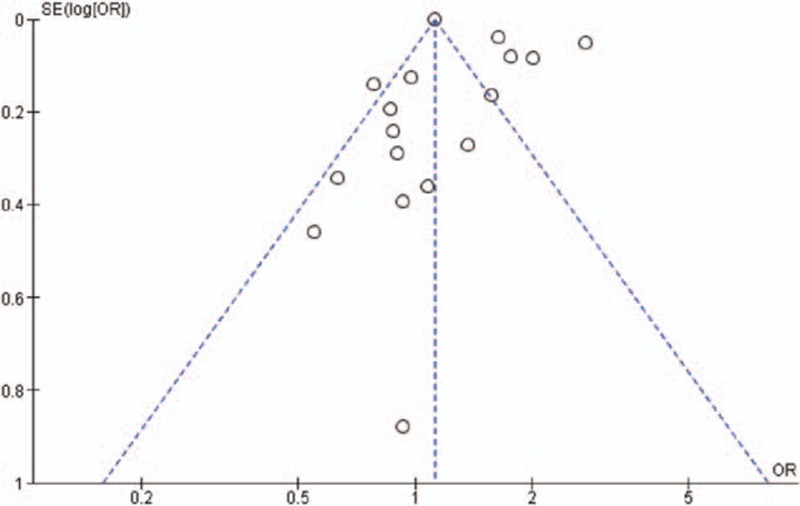
A funnel plot of the overall meta-analysis of metabolic and endocrine comorbidities reflecting publication bias.

Eleven studies^10–20^ were selected by following the eligibility criteria (Figure 1). Overall, these studies analyzed 117,548 asthma patients and 443,948 nonasthma control patients. Average age (mean ± standard deviation; range) of asthma patients was 57.23 ± 11.65 (18.4 ± 17.8 to 76 ± 4.7) years. Gender distribution in the overall population was: 47 ± 7.1 % males and 52.3 ± 7.1 % females). A considerable publication bias was evident from the visual examination of the funnel plots (Figure 2).

Cardiovascular and cerebrovascular comorbidities included arrhythmia, atrial fibrillation, congenital heart disease, congestive heart failure, coronary artery disease, ischemic heart disease, heart failure, myocardial infarction, peripheral artery disease, valve disease, and stroke. Metabolic comorbidities included diabetes mellitus, dyslipidemia, hyperlipidemia, hypertension, hyperthyroidism, hypothyroidism, obesity, and fluid/electrolyte disorders. Neurological and psychiatric comorbidities included autism, anxiety, cerebral palsy, dementia, depression, cognitive impairment, hemipelgia, ottitis, and psychosis.

Gastrointestinal and urinary comorbidities included gastroesophegeal reflux disease, hernia, liver disease, ulcer, and renal disease/failure. Respiratory comorbidities included chronic bronchitis, respiratory infections, allergy rhinitis, chronic obstructive pulmonary disease, and sinusitis. Other comorbidities included cataract, alcohol/drug abuse, dermatitis, anemia, coagulopathy, and acquired immunodeficiency syndrome (AIDS).

**FIGURE 3:**
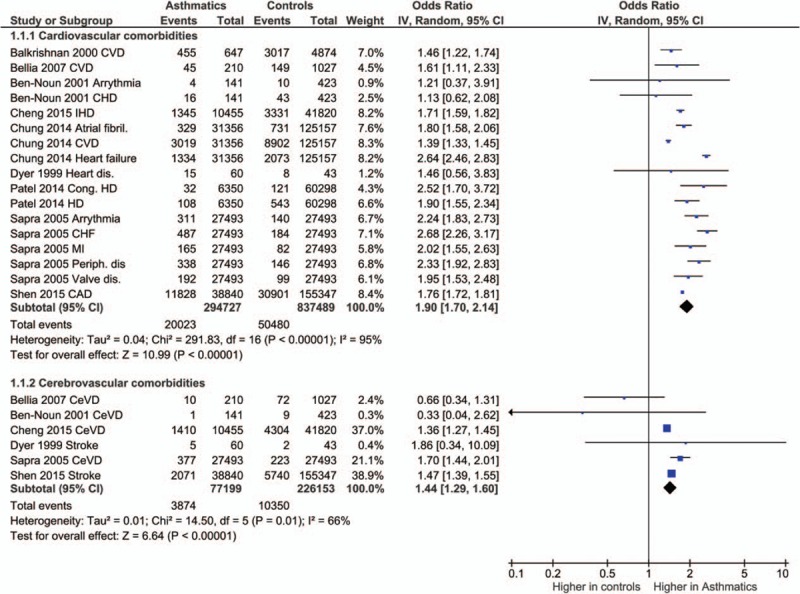
Forest plots showing significantly higher prevalence of cardiovascular and cerebrovascular comorbidities in asthma patients. CAD = coronary artery disease, CeVD = cerebrovascular disease, CHD = coronary heart disease, CHF = congestive heart failure, Cong. HD = congenital heart disease, CVD = cardiovascular disease, dis = disease/disorder, MI = myocardial infarction.

**FIGURE 4:**
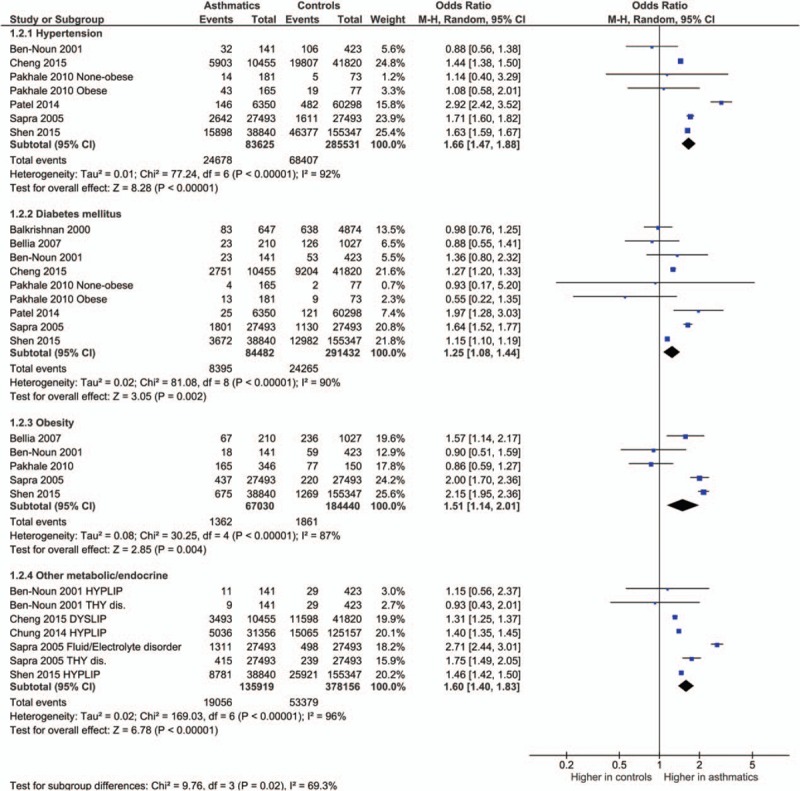
Forest plots showing significantly higher prevalence of metabolic comorbidities in asthma patients. dis. = disease/disorder, DYSLIP = dyslipidemia, HYPLIP = hyperlipidemia, THY = thyroid.

**FIGURE 5:**
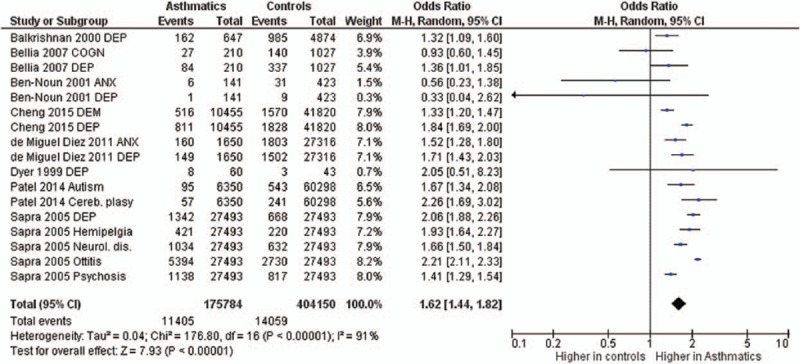
Forest plots showing significantly higher prevalence of neurological and psychiatric comorbidities in asthma patients. ANX = anxiety, COGN = cognitive impairment, DEM = dementia, DEP = depression, dis. = disease/disorder.

**FIGURE 6:**
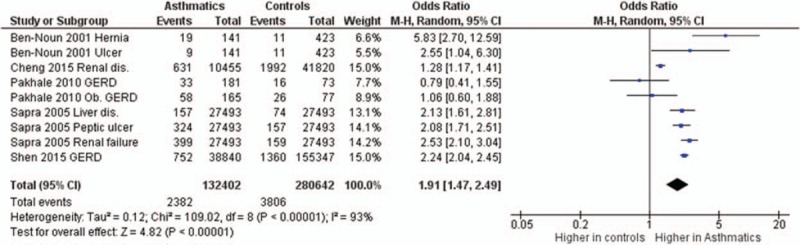
Forest plots showing significantly higher prevalence of gastrointestinal and urinary comorbidities in asthma patients. dis = disease/disorder, GERD = gastro-esophageal reflex disease.

**FIGURE 7:**
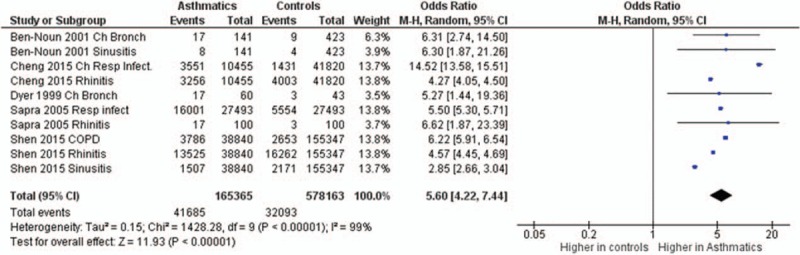
Forest plots showing significantly higher prevalence of respiratory comorbidities in asthma patients. Bronch = bronchitis, ch = chronic, COPD = chronic obstructive pulmonary disease, Infect = infection/s, Resp = respiratory.

**FIGURE 8:**
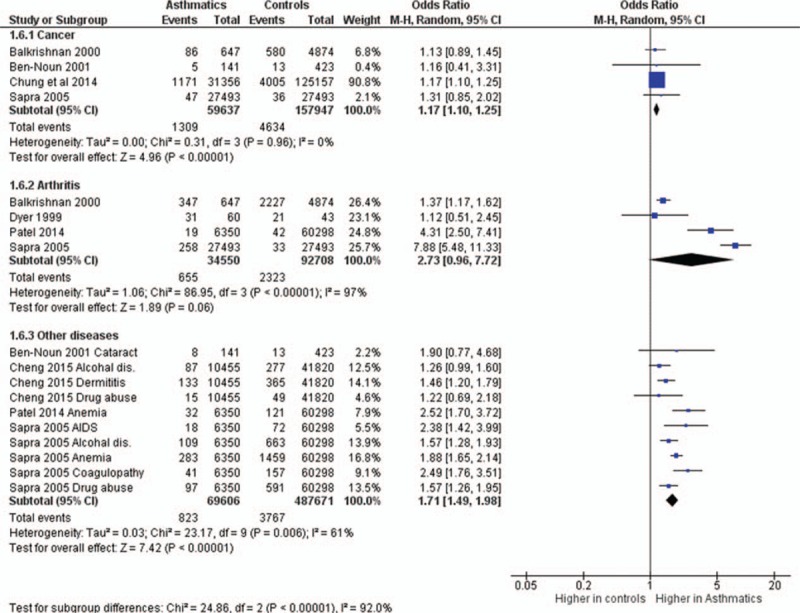
Forest plots showing significantly higher prevalence of cancer and other comorbidities in asthma patients. However, the prevalence of arthritis was not significantly different between asthma and nonasthma patients.

The odds ratios of the prevalence of comorbidities including cardiovascular and cerebrovascular comorbidities (Figure 3), hypertension, diabetes mellitus, obesity, other metabolic, and endocrine comorbidities (Figure 4), neurological and psychiatric comorbidities, gastrointestinal and urinary comorbidities, respiratory comorbidities, cancer, and other some other disease were significantly higher in asthma patients than in nonasthma control patients (Table 1; Figures 5–8). Arthritis was the only disease which was not statistically significantly differently prevalent between asthma and nonasthma patients under the random effects model.

**TABLE 1:**
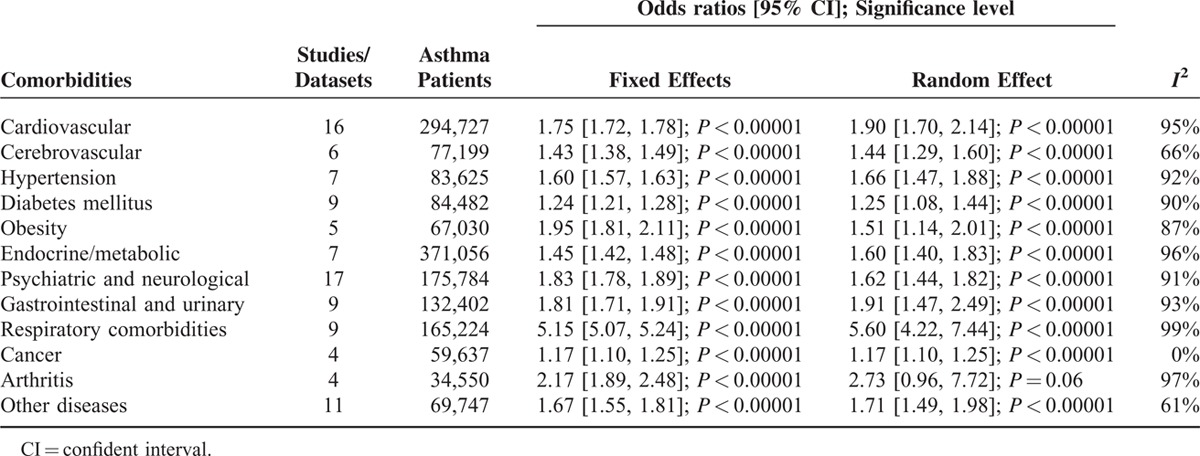
Results of the Meta-Analyses

## DISCUSSION

The present study has compared asthma patients with nonasthma control patients and has found that asthma is associated with significantly higher comorbidities. Almost all types respiratory, cardiovascular, cerebrovascular, metabolic, gastrointestinal, urinary, neurological, and psychiatric, were significantly higher in asthma patients. Only rheumatoid arthritis was found to be statistically indifferent between asthma and nonasthma patients.

Chronic inflammatory conditions appear to be an important factor for the development of comorbidities, for example, asthma is associated with an increased the risk of cardiovascular disease which is attributed to systemic inflammation.^21^ Moreover, in poorly controlled respiratory obstructive conditions, the use of systemic steroids, reduced activity/exercise, and poor sleep can promote comorbidities such as obesity, diabetes, depression, osteoporosis, and pneumonia.^1^

A comorbid condition can also develop or promote other comorbidities. While comparing the comorbid patterns of 292 obese and 383 nonobese asthmatic patients, Shah and Yang^22^ found that the prevalence of diabetes, lipid disorders, and hypertension was significantly higher in obese asthmatics. Moreover, obese patients are found to have about 2.5 times higher risk of developing asthma, asthma is found more severe in obese individuals, and obesity is associated with poor asthma control.^23–25^ Bariatric surgery of morbidly obese patients with asthma has been found to significantly improve airway function and airway hyper-responsiveness besides improving asthma control and systemic inflammation markers.^26^

With aging, the number of comorbid conditions in asthma patients increase. Tsai et al^27^ in a sample population of 1,195,109 asthma patients found that the overall prevalence of 10 selected comorbidities was <1% in patients with under 18 years of age, 3.4% in 18 to 54 years age group, and 12% in patients over 55 years of age. In young individuals, the percentage of asthmatic patients with one kind of comorbidity was 34% and with >1 kinds of comorbidity was 11%.^28,29^ Other studies have also reported significantly higher prevalence of comorbidities in elderly in comparison with nonelderly asthma patients.^30,31^ Recently, Zein et al^32^ have found that after adjusting comorbidities and asthma duration, severity of asthma was significantly higher in individuals >45 years of age.

It is now agreed that asthma in late age is common and relatively less-recognized, underdiagnosed, and undertreated health problem that affects the quality of health and life. It is also speculated that asthma in elderly is causally and phenotypically different from young-age asthma with impact on the diagnosis, assessment, and management.^33^ Although Wardzynska et al^30^ who reported that in elderly asthmatics comorbidities are more prevalent than in nonelderly patients, but apparently this was not having direct impact on asthma control in patients under specialist care, other authors are concerned about potential impacts of comorbidities and their management such as issues related to drug interactions and adverse events resulting from polypharmacy and adherence to therapy.^34^ Gershon et al^6^ who also observed significantly higher comorbidity in asthmatics than in controls, noted that asthma comorbidities were associated with poor asthma control, increased health care utility, and decreased quality of life, but the management of comorbidities could significantly improve the outcomes. Thus, comorbidity should be considered with more emphasis in asthma control strategies and should be an important component of covariate analyses in studies with subjective health outcomes.^35^

Among the foremost limitations of the present study, high statistical heterogeneity representing lower consistency between studies in some comparisons is an important consideration. However, because of the epidemiological nature of the present study, the impact of higher statistical heterogeneity may be less on the overall outcomes. Thus, the availability of more data in future can refine these findings. Another constraint is that majority of the included studies reported selected comorbidities so not all comorbid conditions could be studied here and some comparisons remained underpowered.

## CONCLUSION

Asthma is associated with significantly higher comorbid conditions including cardiovascular and cerebrovascular comorbidities, obesity, hypertension, diabetes, metabolic and endocrine conditions, psychiatric and neurological comorbidities, gut and urinary comorbidities, cancer, and respiratory problems other than asthma. Respiratory comorbidities are found 5 times more prevalent in asthma than in nonasthma patients. Management of comorbidities in asthma control strategies can improve outcomes.
